# The newborn brain is sensitive to the communicative function of language

**DOI:** 10.1038/s41598-022-05122-0

**Published:** 2022-01-24

**Authors:** Bálint Forgács, Tibor Tauzin, György Gergely, Judit Gervain

**Affiliations:** 1 Integrative Neuroscience and Cognition Center, Université de Paris, 45 rue des Saints Pères, 75006 Paris, France; 2 Department of Cognitive Psychology, Institute of Psychology, ELTE Eötvös Loránd University, Izabella u. 46, Budapest, 1064 Hungary; 3 Department of Cognitive Science, Cognitive Development Center (CDC), Central European University (CEU), Október 6. u. 7, Budapest, 1051 Hungary; 4 Dipartimento di Psicologia dello Sviluppo e della Socializzazione – DPSS, Università Padua, Via Venezia 8, 35131 Padua, Italy

**Keywords:** Psychology, Language, Cognitive neuroscience

## Abstract

Recent studies demonstrated neural systems in bilateral fronto-temporal brain areas in newborns specialized to extract linguistic structure from speech. We hypothesized that these mechanisms show additional sensitivity when identically structured different pseudowords are used communicatively in a turn-taking exchange by two speakers. In an fNIRS experiment newborns heard pseudowords sharing ABB repetition structure in three conditions: two voices turn-takingly exchanged different pseudowords (Communicative); the different pseudowords were produced by a (Single Speaker); two voices turn-takingly repeated identical pseudowords (Echoing). Here we show that left fronto-temporal regions (including Broca’s area) responded more to the Communicative than the other conditions. The results demonstrate that newborns’ left hemisphere brain areas show additional activation when various pseudowords sharing identical structure are exchanged in turn-taking alternation by two speakers. This indicates that language processing brain areas at birth are not only sensitive to the structure but to the functional use of language: communicative information transmission. Newborns appear to be equipped not only with innate systems to identify the structural properties of language but to identify its use, communication itself, that is, information exchange between third party social agents—even outside of the mother–infant dyad.

## Introduction

Humans are a highly social species, adapted to acquire language, our species-unique capacity enabling communicative information transmission between cooperative partners. Recent research uncovered neural systems in the newborn brain, mainly in left inferior frontal areas, involving Broca’s area, dedicated to language processing in adults^1^, which are specialized for extracting serial structural regularities from speech^2, 3^. These regions show higher activation for ABB structured pseudowords (“mu-be-be”) than to random ABC controls (“mu-be-ga”), since such repetitions could hint at abstract rule-like structures, indicative of human language^4^. Such activations may reflect early precursors of syntactic abilities emerging already in infancy^5–7^.

A separate line of research demonstrated preverbal infants’ sensitivity to specialized ostensive signals that highlight communicative intentions^8^ and preparedness for ostensive-inferential communication^9^. 10.5-month-old infants attribute agency to unfamiliar entities who produce turn-taking exchanges of variable signal sequences^10^ and 13-month-olds assume that exchanging variable signal sequences—as opposed to repetition of identical signals—transmit information^11^. Importantly, these studies identified two necessary criteria for infants to recognize communicative information transfer. First, identifying two agents, who are taking interactional turns in producing signal sequences. Second, recognizing that the signal sequences exchanged vary sufficiently to potentially transfer novel information between the agents. The latter reflects the information theoretical constraint that by exchanging identical signals it is not possible to transmit information^12^. These two lines of research may demonstrate separate cognitive adaptations, however, recognizing communication and information exchange may provide a key context for acquiring the structural properties of language already from birth.

Here we use functional near-infrared spectroscopy (fNIRS) to test the hypothesis that newborns’ neural systems dedicated to extracting linguistic structure are integrated with their preparedness to recognize the functional use of language for communicative information transmission between agents. We built on a previous fNIRS study^4^ and used sequences of pseudowords that shared an ABB structure to create three conditions. In our main, Communicative condition, a female and a male speaker took turns in uttering different ABB tokens (e.g., female: “ze-pi-pi”—male: “pe-na-na”; male: “sa-lu-lu”—female: “bi-pe-pe”). In the Single Speaker control condition, in each block either a male or a female voice uttered various ABB sequences (e.g., female: “ze-pi-pi”—“pe-na-na”—“mu-fe-fe”), providing the same amount of syllabic variability as the Communicative condition. In the Echoing condition, a male and a female speaker took turns in repeating identical pseudowords continuously (e.g., female: “ze-pi-pi”—male: “ze-pi-pi”; male: “lu-fe-fe”—female: “lu-fe-fe”). We predicted that if newborns are sensitive to both criteria of communicative information exchange, that is, the presence of two speakers and the variability of exchanged tokens, they should respond more strongly to the Communicative condition than to the two control conditions.

## Results

**Figure 1:**
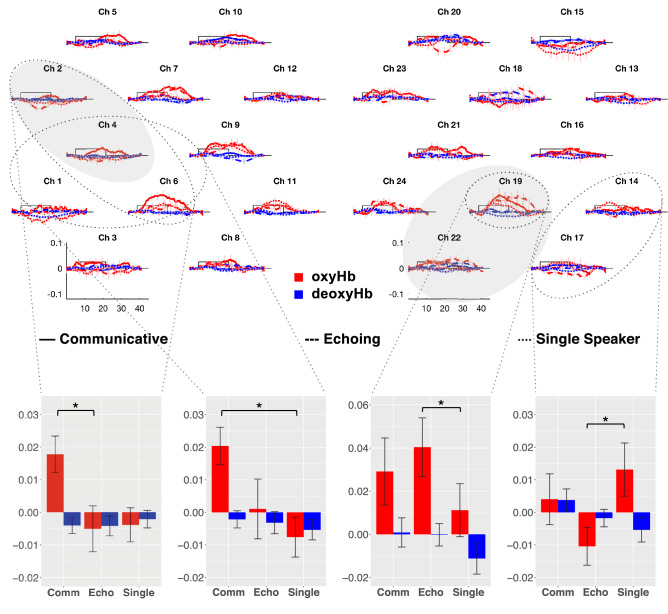
Grand average of oxyHb and deoxyHb. Concentration changes over each channel, averaged across all blocks for all three conditions. Channels are plotted in the layout shown in Fig. 2C. The x-axis represents time in seconds, the y-axis concentration in mmol*mm. The rectangle along the x-axis indicates time of stimulation in seconds. Red and blue colors represent oxyHb and deoxyHb concentrations, respectively. Error bars indicate standard errors of the means. The grey shaded area shows the spatial clusters in which the main ANOVA-based permutation yielded differences across conditions. The bar plots show the significant differences obtained in the clusters (encircled with dotted lines) identified by the follow-up pairwise permutation tests (the third, uncompared condition is shown for convenience).

The hemodynamic responses are shown in Fig. 1. Cluster-based permutation tests^13^ show that the newborn brain responded to all three conditions significantly more than to baseline (Supplementary Fig. 1), as expected on the basis of newborns’ documented preference for repetition-based structures^4^. Importantly, a cluster-based permutation test using an ANOVA with Condition (Communicative, Single Speaker, Echoing) as a within-subject factor over oxyHb concentration changes revealed a significant main effect of Condition over the bilateral fronto-temporal areas, including channels 2 and 4 in the LH (*p* < .001) and channels 19 and 22 in the RH (*p* < .001) (Fig. 1, main panel, clusters shaded in grey). Follow-up permutation tests (Fig. 1 insets for encircled clusters) with pairwise t-tests of the conditions showed a greater oxyHb response to the Communicative than to the Echoing condition in a cluster including channels 2, 4, 6 (*p* < .001). The Communicative condition also evoked stronger responses than the Single Speaker condition in the LH over channel cluster 1, 4, 6 (*p* < .001). Additionally, stronger activity was found for the Single Speaker than for the Echoing condition over the RH channel cluster 14 and 17 (*p* < .001), whereas the opposite pattern was observed in RH channel 19 (*p* < .001), although neither differed from the Communicative condition. A similar analysis over deoxyHb did not show significant results, as is usual with infant fNIRS data.

## Discussion

In an fNIRS experiment we demonstrated that left fronto-temporal areas of the newborn brain show increased activation to structured speech when it is communicatively exchanged by two voices in turn-taking alternation, with the potential for information transmission. Only our Communicative condition met both criteria for communicative information exchange^10, 11, 14^: (A) two agents taking interactive turns, where (B) the exchanged signals contain variability. The Single Speaker condition presented the same sequence of different pseudowords, but lacked turn-taking between two agents, while the Echoing condition involved two speakers, who, however, repeated identical pseudowords with no variation. We found that relative to the other two conditions, the Communicative condition evoked increased activation in the left fronto-temporal areas of newborns' brain, which have been shown to subserve language processing in humans form birth^15^.

The significance of the present finding is that increased neural activation to communication was observed in left fronto-temporal regions, including Broca’s area, which are specialized to respond to linguistic structures, not to processing acoustic features^2, 16^. The very brain region that is activated by sequences with repetitive (ABB) as opposed to random structured pseudowords (ABC)^4, 17, 18^ showed *additional* activation to the ABB baseline of the Single Speaker condition, when varying ABB structured pseudowords were exchanged by two voices in the Communicative condition. This outcome suggests that neural systems dedicated to language acquisition serve not only the extraction of linguistic structures, but recognize the communicative, information transmitting use of language. Importantly, this effect is not simply social: if two agents take turns using linguistic structures interactively but merely repeat each other (Echoing condition), these systems are not activated. Newborns could have shown lower neural activity to turn-taking repetitions of identical linguistic stimuli because perfect predictability and lack of signal variability were not compatible with transmission of novel information.

Even though communication is thought to play a role in various models of language evolution^19, 20^, we did not intend to investigate the origins of language, and the potentially innate mechanisms we identified are not derived from any particular theory of how language evolved. In fact, our results are just as well compatible with language models that are not motivated by its communicative use but by the productivity of thought^21^; such an account is not precluded by models of ostensive inferential communication^22^. Inferential models of communication^9, 23^ suggest that the kind of communication humans engage in is specific to our species^24^, and human infants are indeed sensitive to communicative ostensive-referential cues^8, 25^ including contingent reactivity^26^. In our study, even though all stimuli were perfectly contingent temporally, for which newborns may have a preference^27^, their brain activated less to a perfectly contingent exchange of contents (Echoing) and more to a high-but-imperfect level of contingency, structurally identical yet variable tokens (Communication), as if they sought to extract more information from such imperfectly contingent signal sequences. In a broader sense, Echoing may be taken to be a special kind of communicative exchange (e.g., ritualized greetings), which, in non-continuous form, typically addresses the recipient by expressing communicative intent that the recipient accepts. Yet our results show that newborns have a preference for higher communicative complexity, which enables them to recognize potential information transfer. In sum, no current nativist model appears to predict that any kind of sensitivity to communicational pragmatics or to the possibility of information transfer may be present at birth, over and above recognizing syntax-like structural complexity (ABB). We hope that our results will open the door for future research to approach these intriguing questions.

One possible alternative interpretation of our results could be based on low-level acoustic feature processing, driven by stimulus complexity. Even though, general linguistic complexity was held constant across conditions by the ABB structure, it could be argued that the Communicative condition contained both high speaker variability (like the Echoing condition) and high token variability (like the Single Speaker condition), thus the results reflect the combined effect of these two factors. However, on the basis of our results, such an explanation seems unlikely. Notably, the combined token and speaker variability is identical in the Communicative and the Single Speaker conditions across blocks: not only the number of tokens is the same, but it is either a male or a female voice that can be heard in the Single Speaker blocks. Moreover, it should be assumed that left fronto-temporal areas, which have been identified to track repetitive (ABB) structures and thereby rule-like linguistic complexity, are activated in the Communicative condition over and above the Single Speaker condition exclusively due to the acoustic features of a second speaker. However, acoustic processing takes place in temporal areas, thus if it played a role, we would expect to see it there. In contrast, left inferior frontal areas, including Broca’s area, are sensitive to structural complexity, not to the acoustic features of language^2, 16^. Taken together, if token variability drove our results, there should have been no difference between the Communicative and the Single Speaker conditions, and if speaker variability were the key factor, there should have been no difference between the Communicative and Echoing conditions. Our findings seem to suggest instead that the newborn brain does not carve out the stimulus space the way a low-level acoustic feature processing account would predict.

The Echoing condition evoked stronger responses than the Single Speaker condition in right anterior areas, while the opposite pattern was observed in right mid-temporal areas, but crucially, neither control conditions differed from the Communicative condition. These activations are, indeed, likely to reflect auditory and prosodic processes^28, 29^. Two opposing mechanisms may be at play here—however, both of them are different from the above, complexity account. In the Echoing condition the alternation of the female and male voices creates a variable pitch pattern, which may give rise to enhanced prosodic processing at certain locations, while the repetitions may trigger habituation, resulting in decreased activation at other locations. Different areas of the right hemisphere may be more sensitive to one effect or the other, resulting in the opposite pattern of activations. The Communicative condition, by contrast, shares the pitch alternation pattern with the Echoing and the variability of the pseudowords with the Single Speaker condition, so auditorily it does not considerably differ from either, irrespective of the specific mechanism involved.

Previous studies revealed neural systems in the bilateral fronto-temporal areas of the newborn brain that employ dedicated mechanisms to extract linguistic structure from speech^4, 17^. The present experiment extends these findings by showing that the same neural systems respond with additional activation when exposed to communication, that is, two speakers producing varying pseudowords in turn-taking alteration with the potential for information transmission. While newborns have been reported to engage in turn-taking vocalizations with their mother^30^, our result is the first to show their special responsiveness to third-person communicative exchanges. This result demonstrates an extended range of sensitivity to linguistic stimuli when used for communicative purposes, which is likely to provide special learning opportunities both to acquire the structural properties of language and the information content of communication. Our findings open the door for future research to explore the possibility that humans’ dedicated neural mechanisms for language acquisition may be integrated with systems sensitive for ostensive-inferential communication^9^, which may enable learning the social use of human language rapidly.

## Methods

### Experimental design

**Figure 2:**
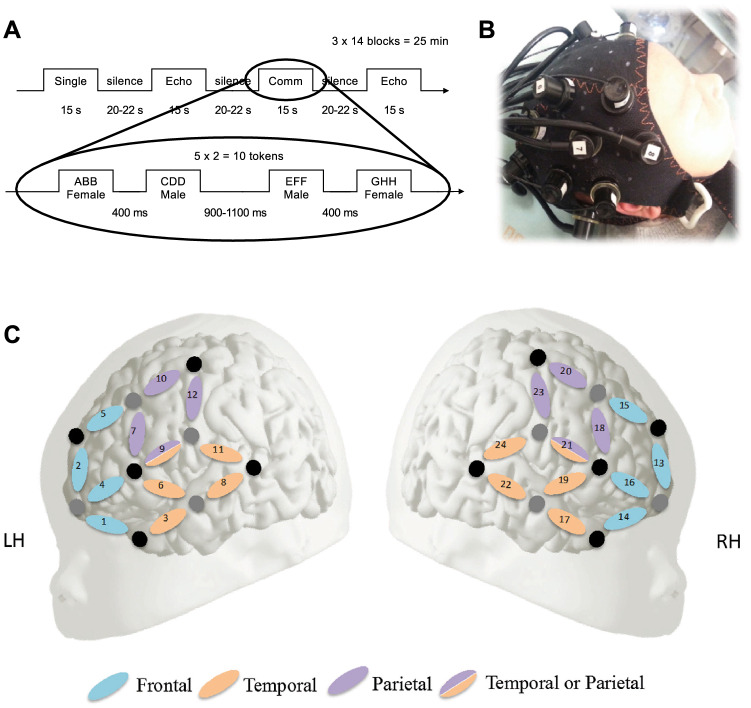
Experimental procedures and channel layout. (**A**) Experimental design. The study was construed in a block design. Blocks of the three experimental conditions and trials within each block were pseudorandomized. (**B**) An fNIRS cap mounted on a newborn. (**C**) Channel layout and spatial localization in the present study, figure adapted from^13^. Permission was granted by parents for publishing image.

The three conditions (Fig. 2A) were presented in 42 blocks (14 per condition, 10 trials each), with the conditions pseudo-randomly interleaved. A block contained 5 turn-taking pairs. Each block lasted ~ 15 s and was followed by a silence of 20–22 s jittered in duration, totaling 25 min. Pseudowords were synthesized using the French fr4 voice of MBROLA for female and fr3 for male voice. The two ABB pseudowords in a turn-taking pair were separated by silences of 400 ms, while the pairs were separated by silences of 900–1100 ms. Block, pseudoword, and speaker order were pseudo-randomized.

### Participants

Twenty-one healthy, full-term, monolingual French newborns (nine female, mean age: 1.76 days, age range: 0–3 days, Apgar score = 10) participated in this study. Nine additional infants were tested, but not included in the final analysis: four did not finish the experiment due to waking up or becoming fussy, and five were excluded because of technical/stimulus error. Newborns were tested while sleeping (Fig. 2B). Parents gave written informed consent to the experiment prior to the testing session. The study was approved by the “Conseil d’évaluation éthique pour les recherches en santé” (CERES) ethics board of Université de Paris, France, and all procedures were performed in accordance with relevant guidelines and regulations.

### Stimuli

A 140 tokens of pseudowords of a repetition-based ABB structure (e.g., “mufefe”) were synthesized using MBROLA (https://github.com/numediart/MBROLA) both with a female (fr4) and a male (fr3) French voice in a monotonous pitch of 200 Hz and a 150 ms length for all phonemes. In the Communication condition one of the voices uttered one pseudowords token, and the other voice responded with a different token (male: ABB—female: CDD; female: EFF—male: GHH; etc.). Question and answer dynamic was achieved by shorter gaps within (400 ms) than between (900–1100 ms) stimulus pairs. In the Echoing condition the first token uttered by the first voice was repeated by the second voice (female: ABB—male: ABB; male: CDD—female: CDD; etc.). In the Single Speaker condition the same temporal dynamics were used for each stimulus pair, but only either the male or the female voice spoke within a block (female: ABB-CDD; EFF-GHH; etc.). All tokens appeared once in the Communication and the Single Speaker conditions and twice in the Echoing condition (the Echoing condition of each two stimulus lists gives a full token list). Each block consisted of 10 tokens, which yielded 14 blocks per condition and 42 in total. One block lasted about 15 s, which was followed by a 20–22 s break to avoid phase-locking. The experiment as a whole took about 25 min. Blocks, pseudowords, and speaker order were pseudo-randomized using Python 2.7 and the experiment was presented using E-prime 2.10.

### Procedure

Newborns were tested lying comfortably sleeping or at rest in their hospital bassinets and listening to the stimuli through loudspeakers at the rear end of their bassinets, while their hemodynamic responses were measured using fNIRS in frontal, temporal and parietal brain areas (Fig. 2B–C), known to be involved in speech processing already at birth^2, 13^. The experiment was carried out at the maternity ward of Robert Debré Hospital, Paris, France, in quiet and dimly lit room with at least one parent present throughout the session. Optical imaging was performed using a NIRScout 816 (16 s–16 d) machine (NIRx Medizintechnik GmbH, Berlin, Germany) providing 24 channels for measuring hemodynamic responses with a source-detector distance of 3 cm. Pulsating LED sequential illumination (5 mW) in two wavelengths (760 nm and 850 nm) were emitted, and the NIRS signal was recorded at 10.4 Hz sampling rate. Twenty-four channels were established by placing four LED sources and five detectors at analogous locations of the two hemispheres, yielding 12 channels on each side (Fig. 1). Optodes were embedded in a cotton cap (Easycap) with a diameter of 36 cm, suitable for newborns, which ensured the standard 3 cm separation for sources and detectors. Channels covered frontal, temporal and parietal brain areas, reported to be involved in speech and language processing by previous studies^4, 13^.

### Statistical analyses

In order to capture the entire time course of hemodynamic responses to stimulation, analyses were run in the 0–15 s time window after stimulus onset, on both oxygenated hemoglobin (oxyHb) and deoxygenated hemoglobin (deoxyHb). Low frequency noise (e.g., drifts in Hb concentration) and high frequency noise (e.g., heartbeat) were removed by band-pass filtering raw data between 0.01 and 0.7 Hz. To exclude movement artifacts, channels were identified in each block with a concentration change greater than 0.1 mmol × mm over a period of 0.2 s (i.e. two samples) and were rejected. If a channel provided useful data only for less than half of the blocks in a condition, it was discarded. Blocks with excluded channels occurred in a non-systematic (e.g., consecutive) pattern. For each block a baseline level of activity was calculated using a linear fit over the 5 s window preceding the onset and 15 s after the end of the block. Fifteen seconds delay was kept to allow for the hemodynamic responses to settle^4, 30^. Statistical analyses were conducted using MATLAB (version R2016b) with dedicated analysis scripts. To reduce the likelihood of false positive results, the permutation tests took into account the autocorrelational properties of the hemodynamic response. The processing steps are described in detail by Abboub et al.^13^.

## Supplementary Information

Supplementary Figure S1.

## Data Availability

Preprocessed data, stimulus randomization, and presentation code are available at https://osf.io/dmyhz/.
