# Supplementary material for: The newborn brain is sensitive to the communicative function of language
Source: Sci Rep. 2022 Jan 24;12:1220. doi: 10.1038/s41598-022-05122-0 (PMC8786876; doi:10.1038/s41598-022-05122-0)
Supplement: Supplementary file 1 — Supplementary Figure S1. [file 41598_2022_5122_MOESM1_ESM.pdf]

Supplementary Information

The Newborn Brain Is Sensitive to the Communicative Function of Language

Bálint Forgács*, Tibor Tauzin, György Gergely, Judit Gervain

*Corresponding author: Bálint Forgács
E-mail: forgacs.balint@ppk.elte.hu
Website: https://sites.google.com/view/balint-forgacs

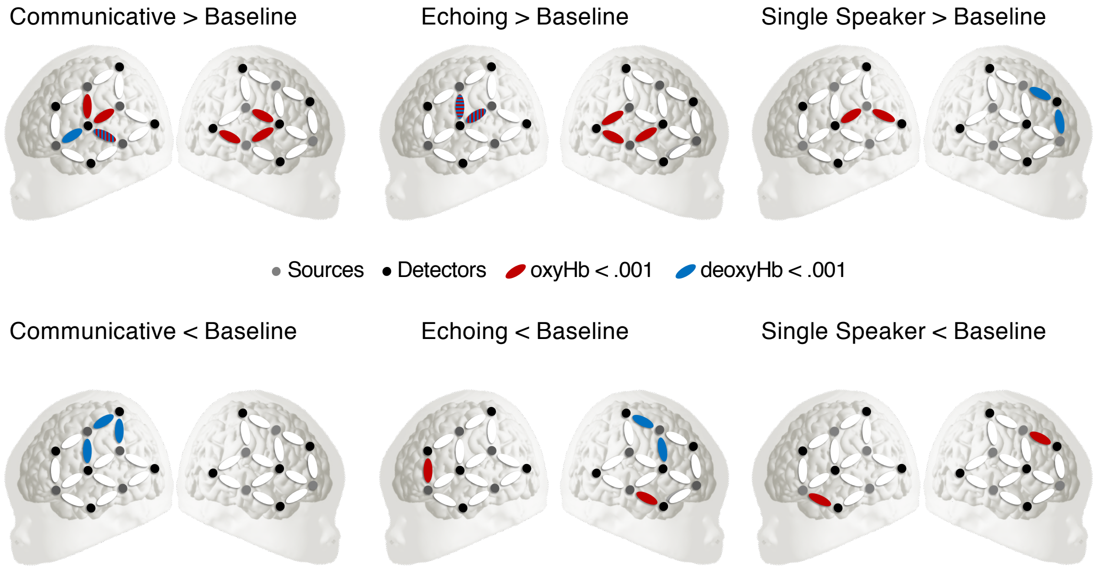

Figure S1. Significant activations, compared to baseline, in the three conditions. Red indicates oxyHb and blue deoxyHb. For deoxyHb results Condition > Baseline means significantly more negative, that is, canonical responses than baseline, while for oxyHb Condition < Baseline means significantly more positive, that is inverted, responses. Channels are plotted in the same layout as in Figure 2C. The neonate brain reacted significantly more to all three conditions than to baseline, primarily in the bilateral temporo-parietal areas.
